# Editorial: Research, Development and Clinical Trials for Peptide-Based Vaccines

**DOI:** 10.3389/fimmu.2022.894989

**Published:** 2022-04-22

**Authors:** Shisong Jiang, Min Gong, Xiao-Ning Xu

**Affiliations:** ^1^ Department of Oncology, University of Oxford, Oxford, United Kingdom; ^2^ Department of Pharmacy, Tianjin Medical University, Tianjin, China; ^3^ Faculty of Medicine, Department of Infectious Disease, Imperial College London, London, United Kingdom

**Keywords:** Peptides, vaccines, epitopes, innate immunity, adoptive immunity, T cells, clinical trials, editorial

From the eradication of smallpox through to the global response to the COVID-19 pandemic, vaccines have been a cornerstone in the fight against infectious diseases in humans and livestock since the 18^th^ century (1). Although vaccines against COVID-19 are unlikely to eradicate the disease in the way that the smallpox vaccine did, they have proved very effective in preventing death and hospitalisation, with enormous societal and economic benefits (2). There is now considerable research effort invested in building on the success of prophylactic vaccines in controlling infectious disease through the development of therapeutic vaccines for chronic diseases such as inflammation and cancer. These approaches are based on down-regulating or up-regulating immune responses for the treatment of inflammatory disease and cancer respectively, through judicious choice of epitopes, adjuvants and mode of the display to the immune system. Though such therapeutic vaccines have shown some early promise, no commercially available products are yet available.

There are six categories of vaccines in use or under development: live attenuated vaccines, inactivated vaccines, subunit vaccines, toxoid vaccines, viral vector-based vaccines, and nucleic acid (DNA or RNA) vaccines (3). In each case, there are multiple examples of vaccines approved for human use. Despite the different modes of delivery of the antigenic payload in these six classes, their common goal is to stimulate one or all of the following types of immune responses: 1) innate immunity, 2) antibody (humoral) immunity, and 3) T cell immunity.

Peptide-based vaccines are a particular type of subunit vaccine usually characterised by a focus on short sequences encompassing single epitopes, normally produced by chemical synthesis. Peptide vaccines have a number of advantages including better defined and more specific immune responses; a good safety profile; simple manufacture and relatively fast drug development (4). Moreover, the sequences of peptide vaccines can be converted into nucleic acids, so they are easily turned into nucleic vaccines or vector-based vaccines. To make peptide vaccines more effective and suitable for industrial manufacture, new forms of peptide-based vaccines are proposed beyond single epitope and beyond chemical synthesis, for example, synthetic long peptide vaccines and recombinant overlapping peptide vaccines (5–7).

In this collection of eleven articles for Frontiers in Immunology titled “Research, Development, and Clinical Trials for Peptide-based Vaccines” we cover key aspects of peptide vaccine development including epitope selection and vaccine design, conjugation methods and adjuvants, the balance between antibody and T cell immunity, and design of clinical trials.

## Design of Peptide Vaccines and Use of Epitopes

The key starting point for design of peptide vaccines is the choice of epitopes that stimulate humoral and/or T cell immunity. There are several ways of identifying the epitopes.

### Overlapping Synthetic Peptides

Jiang et al. used overlapping synthetic peptides covering the target sequence as a library for screening. In this case, they obtained antisera from SARS-CoV-2 receptor binding domain (RBD)-immunised pigs or mice. The antisera were incubated with overlapping peptides covering the RBD to identify those carrying B cell epitopes. The authors then showed that these peptides were immunogenic in their own right and capable of stimulating neutralising antibodies. However, an obvious caveat in the use of this overlapping peptide approach is that it will miss non-sequential conformational epitopes which may play an important role in neutralising targets (8).

### Computer Algorithm/Database

Epitopes can be predicted by computer software. Gong et al. picked their potential HLA-DR1 restricted, TB-targeted T cell epitopes from the Immune Epitope Database (IEDB, https://www.iedb.org/) which they then validated using an ELISPOT assay. Instead of using the pooled peptides as a vaccine, they produced a recombinant poly-epitope in which the relevant peptides were joined *via* a flexible linker (GGGGS). In humanised mice, the poly-epitope vaccine generated strong cellular immunity which protected mice from TB infection. The obvious advantage of linking epitopes in this way (over the use of pooled peptides) is that there is only a single product, greatly simplifying quality control during manufacture and regulatory compliance.

### Neoantigens: Sequencing + Computer Algorithm

Cancer therapeutic vaccines depend on the identification of tumour-specific neoantigens that can be targeted with peptide-based vaccines. Chen et al. combined next generation sequencing (NGS) with bioinformatics and epitope prediction algorithms to design personalised peptide vaccines. Vaccines designed such way have been tested in seven pancreatic cancer patients with promising results (see clinical study section below).

### Processed Pathogens

A novel epitope mapping approach is described by D’haeseleer et al. The authors obtained antisera from mice that had been immunised with a pathogen. The antisera were conjugated to beads which were incubated with trypsinised pathogen as a peptide library. Peptides retained by the beads were subsequently eluted and identified by mass spectroscopy. They validated epitopes identified in this way for two bacterial pathogens (*Francisella tularensis* and *Burkholderia pseudomallei*) and showed that they were immunogenic. Interestingly, they compared their approach with that of computer-based epitope mapping and found that it was more effective at identifying experimentally validated epitopes, even though some computer-picked epitopes gave higher *in silico* scores. However, a necessary limitation of the approach is that it will miss epitopes that carry a tryptic cleavage site.

### Recombinant Overlapping Peptides

Zhang et al. used recombinant overlapping peptides (ROPs) from human papilloma virus (HPV) type 16 E7 (ROP-HPV16-E7) as a T cell stimulating agent. Overlapping peptides avoid the boundary problem and must therefore contain all possible T cell epitopes for different MHC phenotypes. As a result, they stimulate broad T cell immunity promiscuously. As ROPs are made as a single protein product in *E.coli*, they benefit from a straightforward manufacturing process and offer a comparatively simple path through preclinical development to regulatory approval.

## Conjugation and Adjuvant of Peptide Vaccines

In addition to epitopes capable of stimulating B cells, CD4^+^ and CD8^+^ T cells, peptide vaccines need strong adjuvants to stimulate innate immunity. Rossi et al. describe results with vaccines based on cell penetrating peptide carrying HPV or ovalbumin (OVA) T cell epitopes combined with stimulator of interferon gamma gene agonist (STINGa) as an adjuvant to elicit a potent innate inflammatory response. This strategy stimulated both CD8^+^ and Th1 CD4^+^ T cell responses while inhibiting Treg response. The vaccine was effective in prolonging survival in TC-1 tumour inoculated mice.

Calzas et al. describe a vaccine formulation containing weak but conserved antigens from influenza virus combined with a nanoring comprising elements from respiratory syncytial virus. This ‘nanoring’ stimulates innate immunity - possibly through TLR5. The vaccine is given *via* the mucosal route and induced strong humoral and cellular immune responses. The vaccine protected mice (but not chickens) from viral challenge.

## Clinical Studies of Peptide Vaccines

Three of the above articles involved clinical studies. Two of these addressed HPV infection and both show the importance of T cell immunity in recovery and viral clearance. Shibata et al. describe one case in detail emerging from the Phase I clinical trial of their peptide vaccine (PepCan). The patient, who had a high-grade cervical squamous intraepithelial lesion, received four doses of the peptide vaccine at 4-weeks’ interval. While Treg and Th2 cytokine levels remained unchanged, Th1 cytokine levels were enhanced significantly. At twelve weeks (end of the follow-up), the intraepithelial lesion had completely disappeared, potentially due to this enhanced T cell response. Moreover, using single cell sequencing, the authors showed specific CD3^+^ T cell clonal expansion both systemically and at the lesion site.

In the second HPV-related clinical cohort study, Zhang et al. followed up 131 HPV16-infected patients for 12 months and found that HPV specific T cell immunity is important for the viral clearance.

In the final clinical trial report, Chen et al. investigated the safety and immunogenicity of a neoantigen-based vaccine in seven pancreatic cancer patients. The authors showed that the vaccine was safe and induced both CD4^+^ and CD8^+^ T cell responses. One patient exhibiting significant expansion of a reactive T cell clone survived for 21 months, considerably in excess of the expected 6-month average for patients with this stage of disease.

## Conclusion

Vaccines against infectious disease have to date proved much more successful than vaccines against cancer, essentially because pathogens are non-self and present a much larger immunological target which stimulate strong innate, humoral and cellular immunity. Nonetheless, significant progress has been made in the identification of antigens other than foreign pathogens, e.g., neoantigens and tumour associated antigens (TAA) that can be exploited by vaccine technology, particularly using peptide vaccines. These advances, together with recent developments in the clinical application of IO agents, offer the prospect of personalised combination therapies that could be transformative in the treatment of many cancers.

Note that the research articles collected in this Research Topic are heavily focused on the design of peptide vaccines. For a border perspective on peptide vaccines especially cancer peptide vaccines, see the review article by Stephens et al.

## Author Contributions

SJ, MG and XNX drafted this article. All authors contributed to the article and approved the submitted version.

## Funding

SJ is funded by Innovate UK, grant number 104992 & 133783, and by grants from the CBI and Oxford Vacmedix UK Ltd. MG is supported by Chinese National Natural Science Funding program (81771221, 81870967 and 82071384). The funders were not involved in the study design, collection, analysis, interpretation of data, the writing of this article or the decision to submit it for publication.

## Conflict of Interest

SJ and XNX are founder or consultant of Oxford Vacmedix UK Ltd which develops peptide-based vaccines.

The remaining author declares that the research was conducted in the absence of any commercial or financial relationships that could be construed as a potential conflict of interest.

## Publisher’s Note

All claims expressed in this article are solely those of the authors and do not necessarily represent those of their affiliated organizations, or those of the publisher, the editors and the reviewers. Any product that may be evaluated in this article, or claim that may be made by its manufacturer, is not guaranteed or endorsed by the publisher.
